# A randomised trial to evaluate the immunogenicity, reactogenicity, and safety of the 10-valent pneumococcal non-typeable *Haemophilus influenzae*protein D conjugate vaccine (PHiD-CV) co-administered with routine childhood vaccines in Singapore and Malaysia

**DOI:** 10.1186/1471-2334-14-530

**Published:** 2014-10-02

**Authors:** Fong Seng Lim, Mia Tuang Koh, Kah Kee Tan, Poh Chong Chan, Chia Yin Chong, Yeo Wee Shung Yehudi, Yee Leong Teoh, Fakrudeen Shafi, Marjan Hezareh, Kristien Swinnen, Dorota Borys

**Affiliations:** National Healthcare Group Polyclinics, 3 Fusionopolis Link #03-08, Nexus@one-north, Singapore, 138543 Singapore; Departement of Pediatrics, University Malaya Medical Centre, Jalan University, 59100 Kuala Lumpur, Malaysia; Departement of Pediatrics, Tuanku Ja’afar Hospital, Jalan Rasah, 70300 Seremban, Negeri Sembilan, Malaysia; University Children's Medical Institute, National University Hospital, Singapore, 119260 Singapore; KK Women and Children’s Hospital, 100 Bukit Timah Road, Singapore, 229899 Singapore; GlaxoSmithKline Vaccines, 150 Beach Road, #22-00, Gateway West, Singapore, 189720 Singapore; Current affiliation: Goal Consultancy Asia Pacific, Singapore, Singapore; GlaxoSmithKline Pharmaceuticals, 5 Embassy Links, SRT Road, Bangalore, 560 052 India; Vaccine Discovery and Development, GlaxoSmithKline Vaccines, 20 Avenue Fleming, 1300 Wavre, Belgium

**Keywords:** Pneumococcal conjugate vaccine, Immunogenicity, Safety, Non-typeable *Haemophilus influenzae*, Infant, Toddler, Singapore, Malaysia

## Abstract

**Background:**

The immunogenicity, reactogenicity, and safety of the 10-valent pneumococcal non-typeable *Haemophilus influenzae* protein D conjugate vaccine (PHiD-CV) co-administered with routine childhood vaccines were evaluated among infants from Singapore and Malaysia, where PHiD-CV has been licensed.

**Methods:**

In the primary vaccination phase, 298 infants from Singapore and 168 infants from Malaysia were randomised to receive the Phase III Clinical (Clin) or the Commercial (Com) lot of PHiD-CV at 2, 3, and 5 months of age. In the booster vaccination phase, 238 toddlers from Singapore received one dose of the PHiD-CV Commercial lot at 18–21 months of age. Immune responses to pneumococcal polysaccharides were measured using 22F-inhibition enzyme-linked immunosorbent assay (ELISA) and functional opsonophagocytic activity (OPA) assay and to protein D, using ELISA.

**Results:**

Immune responses induced by primary vaccination with the PHiD-CV Commercial lot were non-inferior to the Phase III Clinical lot in terms of adjusted antibody geometric mean concentration (GMC) ratios for each vaccine pneumococcal serotype and protein D. For each vaccine pneumococcal serotype, ≥93.6% and ≥88.5% of infants from Malaysia and Singapore had post-primary vaccination antibody concentrations ≥0.2 μg/mL and OPA titres ≥8, in the Clin and Com groups, respectively. For each vaccine pneumococcal serotype, ≥60.8% and ≥98.2% of toddlers from Singapore had pre- and post-booster antibody concentrations ≥0.2 μg/mL, in the Clin and Com groups, respectively. All children, except one, had measurable anti-protein D antibodies and the primary and booster doses of the co-administered vaccines were immunogenic. The incidence of each grade 3 solicited symptom was ≤11.1% in both study phases. No serious adverse events considered causally related to vaccination were reported throughout the study.

**Conclusions:**

PHiD-CV given as three-dose primary vaccination to infants in Singapore and Malaysia and booster vaccination to toddlers in Singapore was shown to be immunogenic with a clinically acceptable-safety profile.

This study has been registered at http://www.clinicaltrials.govNCT00808444 and NCT01119625.

**Electronic supplementary material:**

The online version of this article (doi:10.1186/1471-2334-14-530) contains supplementary material, which is available to authorized users.

## Background

*Streptococcus pneumoniae* is responsible for invasive diseases, which cause significant morbidity and mortality worldwide [1]. The incidence of invasive pneumococcal disease (IPD) is especially high in Asia, where children younger than 5 years old are the most severely affected [2–5]. In Singapore, the incidence of IPD reached 15.2 per 100,000 children <5 years of age in 2008–2010; the most common serotypes were serotypes 6B, 19A, 14, and 23F [6]. In Malaysia, there is limited information on the incidence of IPD, although a previous study suggested that the incidence of pneumococcal meningitis reached 8.6 per 100,000 children <5 years of age in 2004–2006 [7]. The most common serotypes in Malaysia in 2008–2009 were serotypes 19F, 6B, 19A, and 14 [6, 8]. In both countries, emergence of antimicrobial resistant *S. pneumoniae* isolates is a major health concern [3, 8–14].

Prevention of pneumococcal infections through vaccination remains the best strategy to reduce the incidence of IPD. A 10-valent pneumococcal non-typeable *Haemophilus influenzae* (NTHi) protein D conjugate vaccine (PHiD-CV; *Synflorix*™, GlaxoSmithKline Vaccines), which contains serotypes 1, 4, 5, 6B, 7F, 9V, 14, 18C, 19F, and 23F, was licensed in Singapore in March 2010 and in Malaysia in September 2009. PHiD-CV has been shown to be immunogenic and well-tolerated when co-administered with commonly used paediatric vaccines in infants and toddlers [15–22]. Since eight of the ten pneumococcal serotypes included in PHiD-CV are conjugated to NTHi protein D, this vaccine has the potential to provide additional protection against NTHi diseases [23].

This study compared the immunogenicity, reactogenicity, and safety of three-dose primary vaccination with a Phase III Clinical lot or a Commercial lot of PHiD-CV co-administered with routine childhood vaccines in infants from Singapore and Malaysia. The immunogenicity and safety of a booster dose of the PHiD-CV Commercial lot were also evaluated in toddlers from Singapore.

## Methods

### Study design

This Phase III study comprised a primary vaccination phase conducted in three centres in Singapore and two centres in Malaysia between January 2009 and November 2009, and a booster vaccination phase conducted in the three Singaporean centres between July 2010 and February 2011. The booster vaccination phase did not encompass Malaysia since PHiD-CV obtained registration in Malaysia in September 2009 and booster vaccination could thus be offered outside clinical trial settings.

In the primary vaccination phase, infants were randomised (1:1) to two parallel groups to receive three doses of the PHiD-CV Phase III Clinical (Clin group) or Commercial lot (Com group) at 2, 3, and 5 months of age. PHiD-CV was co-administered with diphtheria, tetanus, acellular pertussis-hepatitis B virus-inactivated poliovirus, and *H. influenzae* type b vaccine (DTPa-HBV-IPV/Hib) in Malaysia at 2, 3, and 5 months of age and in Singapore at 2 and 5 months of age and with DTPa-IPV/Hib in Singapore at 3 months of age. All infants received two doses of a human rotavirus (HRV) vaccine at 2 and 3 months of age. The primary vaccination phase was double-blinded.

In the booster vaccination phase, all toddlers from Singapore received a booster dose of the PHiD-CV Commercial lot co-administered with DTPa-IPV/Hib at 18–21 months of age. Thus, the booster vaccination phase was conducted in an open-label manner.

The study was conducted in accordance with Good Clinical Practice guidelines and the Declaration of Helsinki. The protocol and associated documents were reviewed and approved by the Medical Research & Ethics Committee of the Ministry of Health in Malaysia and the Medical Ethics Committee of University Malaya Medical Centre and the Domain-Specific Review Board of the National Healthcare Group in Singapore. Written informed consent was obtained before enrolment from the parents or legally acceptable representatives of each child. This study has been registered at http://www.clinicaltrials.gov NCT00808444 and NCT01119625. A protocol summary is available at http://www.gsk-clinicalstudyregister.com (GSK study IDs 111654 and 113266).

### Study objectives

The primary objectives were to demonstrate the comparability of the immune response induced by three-dose primary vaccination with the Commercial lot versus the Phase III Clinical lots of PHiD-CV in infants from Malaysia and Singapore, and to assess the persistence of the antibodies induced by both PHiD-CV lots up to the booster vaccination in toddlers from Singapore. Secondary objectives included the evaluation of the immunogenicity, safety, and reactogenicity of PHiD-CV and the co-administered vaccines after primary and booster vaccinations.

### Study participants

Eligible participants were healthy infants from Malaysia and Singapore aged 6–12 weeks at the time of the first vaccination, who were born after a gestation period of between 36 and 42 weeks. For the booster vaccination phase, eligible participants were healthy toddlers aged 18–21 months at the time of the booster vaccination, who had received three PHiD-CV doses in the primary vaccination phase in Singapore.

Children were excluded from participation if they were concurrently participating in another clinical study; were immunosuppressed; had used investigational products within 30 days pre-vaccination; had previously received blood products; had previous vaccination against or history of diphtheria, tetanus, pertussis, poliomyelitis, hepatitis B, Hib, or pneumococcal disease; or had allergic disease or reactions likely to be exacerbated by the vaccines, history of neurological disorders or seizures, major congenital defects, or a serious chronic illness.

### Vaccines

The Commercial and Phase III Clinical lots of PHiD-CV had the same composition, which has been previously described [21]. They were administered intramuscularly in the right thigh in infants and the right deltoid or thigh in toddlers. All co-administered vaccines were manufactured by GlaxoSmithKline Vaccines. DTPa-IPV/Hib (*Infanrix*™-IPV/Hib) and DTPa-HBV-IPV/Hib (*Infanrix*™ *hexa*) were administered intramuscularly in the left thigh in infants and the left deltoid or thigh in toddlers. The live attenuated HRV vaccine (*Rotarix*™) was administered orally.

### Immunogenicity assessment

Blood samples were collected one month post-primary vaccination and before and one month after the booster vaccination. Antibody concentrations against vaccine pneumococcal serotypes and cross-reactive serotypes were measured by 22F-inhibition enzyme-linked immunosorbent assay (ELISA) as described previously (cut-off: 0.05 μg/mL) [24, 25]. Percentages of children with antibody concentrations ≥0.2 μg/mL were determined. Antibody concentrations of 0.2 μg/mL measured by 22F-inhibition ELISA are equivalent to antibody concentrations of 0.35 μg/mL measured by the World Health Organization reference ELISA without 22F-inhibition, which is the threshold used for comparisons of immune responses induced by different pneumococcal conjugate vaccines [25, 26]. In the primary vaccination phase, opsonophagocytic activity (OPA) was measured by a pneumococcal killing assay using a HL 60 cell line (cut-off titre: 8) [27, 28]. Antibodies against NTHi protein D were measured by an in-house ELISA using a recombinant non-lipidated form of protein D as coating material. Anti-protein D antibodies, which are bound to protein D antigens adsorbed on polystyrene plates, are detected using an anti-human-IgG peroxidase labelled antibody followed by the addition of tetramethylbenzidine substrate. Anti-protein D antibody concentrations were expressed in ELISA units per mL (EL.U/mL), and the assay cut-off was 100 EL.U/mL.

Infants were sub-randomised (1:1) to two subsets for the analysis of immune responses to co-administered vaccines. Immune responses were measured by ELISA [29–34], except for the three poliovirus types, which were measured by a virus microneutralisation test [35]. All laboratory analyses were performed at GlaxoSmithKline (Rixensart, Belgium) or in laboratories designated by GlaxoSmithKline Vaccines. Upon discovery of an anti-hepatitis B assay specificity issue, a risk was identified that children whose post-vaccination titres were initially measured between 10 and 100 mIU/mL might have been informed of an incorrect seroprotection status. Retesting was performed as an urgent precaution using *Immulite*™, a commercial assay used for routine testing at Ghent University Hospital, Belgium.

### Safety and reactogenicity assessment

Solicited local (injection sites pain, redness, and swelling) and general (irritability, drowsiness, fever [rectal/axillary temperature ≥38.0°C/37.5°C], and loss of appetite) symptoms and antipyretic use were recorded during a four-day post-vaccination period. In the primary vaccination phase, vomiting and diarrhoea were also recorded for four days post-vaccination. Unsolicited adverse events (AEs) were recorded for 31 days post-vaccination. The intensity of each symptom was graded on a three-grade scale. Grade 3 was reported for pain if infants/toddlers cried when the limbs were moved or if the limbs were spontaneously painful; redness and swelling, if diameters were >30 mm; fever, if rectal/axillary temperature was >40.0°C/39.5°C; loss of appetite, if infants/toddlers did not eat at all; irritability, if infants/toddlers cried and could not be comforted; diarrhoea, if infants had ≥6 looser than normal stools/day; vomiting, if infants had ≥3 episodes/day; and all other AEs, if they prevented normal activity.

Serious adverse events (SAEs) were recorded throughout the study and were defined as events that were life-threatening, required hospitalisation or prolongation of hospitalisation, or resulted in disability, incapacity, or death. As per protocol, all solicited local symptoms were considered causally related to vaccination. Causality of all other AEs was assessed by investigators.

### Statistical analyses

Safety analyses were performed on the primary and booster total vaccinated cohorts. Immunogenicity analyses were performed on the primary and booster according-to-protocol (ATP) immunogenicity cohorts and on the ATP persistence cohort, which included all infants/toddlers who met all eligibility criteria, complied with protocol-defined procedures, and for whom antibody assay results were available.

Antibody geometric mean concentrations (GMCs), OPA geometric mean titres (GMTs), and percentages of infants/toddlers with concentrations or titres above pre-specified cut-offs/thresholds were calculated with 95% confidence intervals (CIs). GMCs and GMTs were calculated by taking the anti-log of the mean of the log antibody concentration/titre transformations. Antibody concentrations/titres below assay cut-offs were given an arbitrary value of half the cut-off.

Non-inferiority of the Commercial versus the Phase III Clinical lot was demonstrated if the upper limit (UL) of the two-sided 95% CI (calculated using an ANOVA model [pooled variance] adjusting for multi-country effect) on adjusted GMC ratios (Clin over Com group) for antibodies against vaccine pneumococcal serotypes and protein D was below 2.

In the primary vaccination phase, the target sample size was 460 enrolled infants to obtain ≥400 evaluable infants. When comparing both PHiD-CV lots, 200 evaluable infants per group would provide at least 98% power under equal mean or 85% power in case of 1.2-fold decrease in GMCs to show non-inferiority of the Commercial lot compared to the Phase III Clinical lot with respect to adjusted antibody GMC ratios for vaccine pneumococcal serotypes and protein D. In the booster vaccination phase, the target sample size was 298 enrolled toddlers, taking into account the actual enrolment in the primary vaccination phase in Singapore.

Incidences of solicited and unsolicited AEs were calculated with exact 95% CIs. SAEs and withdrawals due to AEs were described in detail. Non-overlapping two-sided 95% CIs were used to highlight potential group differences, which should be interpreted with caution.

Statistical analyses were performed using Statistical Analysis System (SAS® software, SAS Institute Inc., Cary, NC, United States) version 9.2 and SDD (i.e. SAS Drug and Development) web portal version 3.5.

## Results

### Study population

**Figure 1:**
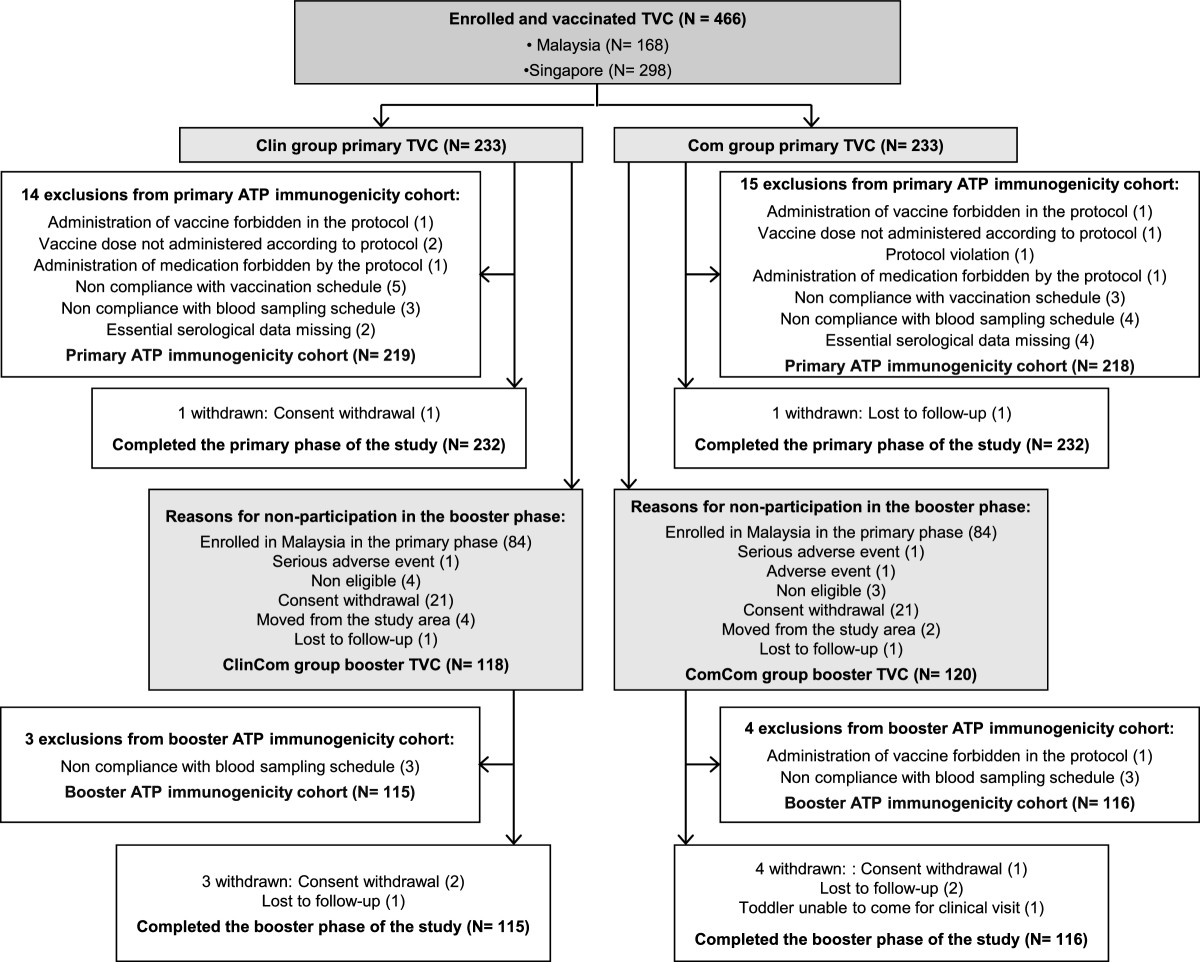
**Trial profile.** Clin = group of infants from Malaysia and Singapore who received the Phase III Clinical lot of PHiD-CV in the primary vaccination phase. Com = group of infants from Malaysia and Singapore who received the Commercial lot of PHiD-CV in the primary vaccination phase. ClinCom = group of toddlers from Singapore primed with the Phase III Clinical lot of PHiD-CV who received the Commercial lot of PHiD-CV in the booster vaccination phase. ComCom = group of toddlers from Singapore primed with the Commercial lot of PHiD-CV who received the Commercial lot of PHiD-CV in the booster vaccination phase.

A total of 466 infants were enrolled and 464 infants completed the primary vaccination phase (Figure 1). 238 toddlers from Singapore were included in the booster vaccination phase and 231 toddlers completed the study. The demographic characteristics of the infants included in the primary ATP immunogenicity cohort were comparable in both groups and were consistent with those of the toddlers from Singapore included in the booster ATP immunogenicity cohort (Table 1).

**Table 1:** **Demographic characteristics of the study participants (primary and booster ATP immunogenicity cohorts)**

Primary vaccination phase		Clin group	Com group
		N = 219	N = 218
**Age**	Mean age ± SD (weeks)	7.3 ± 1.35	7.3 ± 1.31
	Age range (weeks)	6–11	6–11
**Gender**	Female n (%)	108 (49.3)	95 (43.6)
	Male n (%)	111 (50.7)	123 (56.4)
**Race**	Asian – South East Asian heritage n (%)	217 (99.1)	217 (99.5)
	Asian – Central/South Asian heritage n (%)	1 (0.5)	0 (0.0)
	Asian – East Asian heritage n (%)	1 (0.5)	1 (0.5)
**Booster vaccination phase**		**ClinCom group**	**ClinCom group**
		**N = 115**	**N = 116**
**Age**	Mean age ± SD (months)	18.8 ± 0.84	18.9 ± 0.85
	Age range (months)	18–21	18–21
**Gender**	Female n (%)	59 (51.3)	48 (41.4)
	Male n (%)	56 (48.7)	68 (58.6)
**Race**	Asian – South East Asian heritage n (%)	114 (99.1)	115 (99.1)
	Asian – East Asian heritage n (%)	1 (0.9)	1 (0.9)

ATP = according to protocol.

Clin = group of infants from Malaysia and Singapore who received the Phase III Clinical lot of PHiD-CV in the primary vaccination phase.

Com = group of infants from Malaysia and Singapore who received the Commercial lot of PHiD-CV in the primary vaccination phase.

ClinCom = group of toddlers from Singapore primed with the Phase III Clinical lot of PHiD-CV who received the Commercial lot of PHiD-CV in the booster vaccination phase.

ComCom = group of toddlers from Singapore primed with the Commercial lot of PHiD-CV who received the Commercial lot of PHiD-CV in the booster vaccination phase.

N = number of participants.

n (%) = number (percentage) of participants with the specified characteristic.

SD = standard deviation.

Range = minimum – maximum.

### Immunogenicity

#### Pneumococcal vaccine antigens

**Figure 2:**
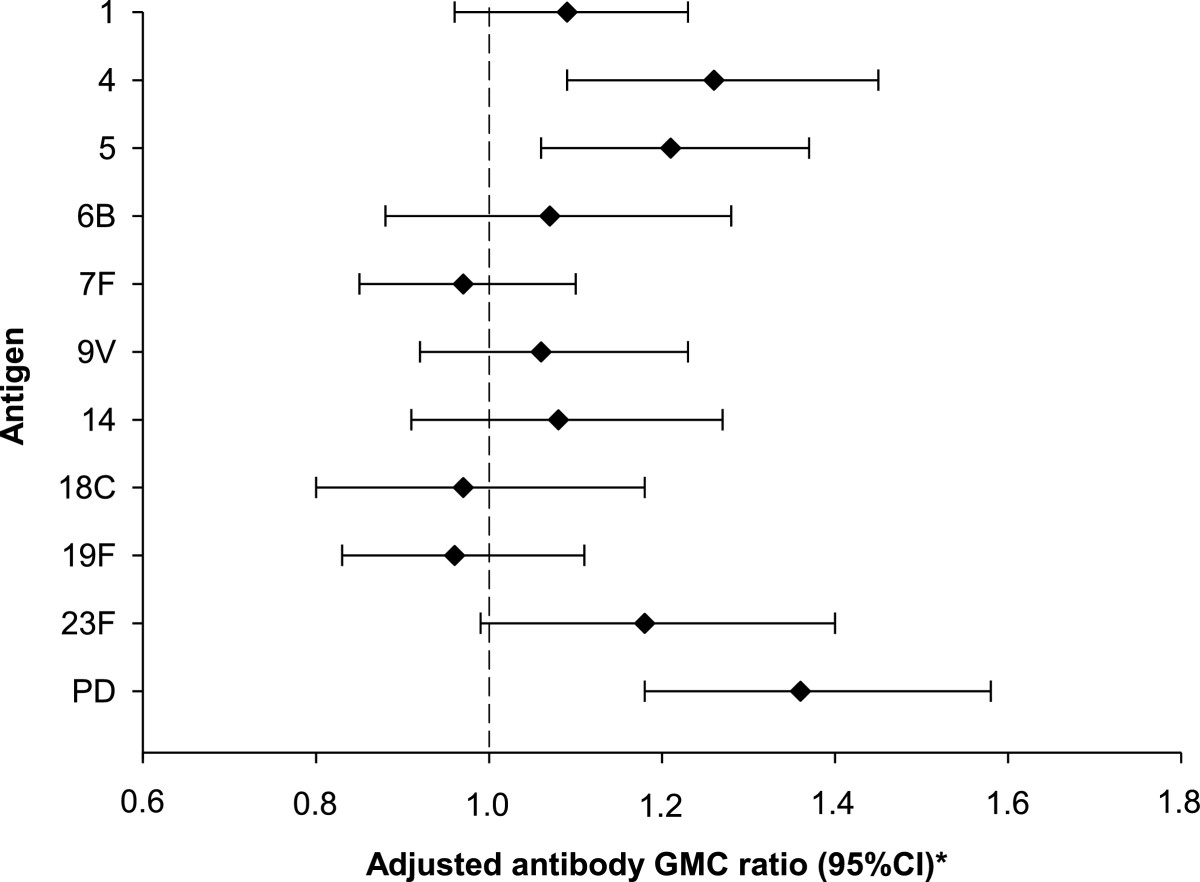
**Adjusted antibody GMC ratios between the Clin and the Com groups for the 10 vaccine pneumococcal serotypes and protein D at one month post-primary vaccination (primary ATP immunogenicity cohort).** Clin = group of infants from Malaysia and Singapore who received the Phase III Clinical lot of PHiD-CV in the primary vaccination phase. Com = group of infants from Malaysia and Singapore who received the Commercial lot of PHiD-CV in the primary vaccination phase. Adjusted antibody GMC ratio = ratio of the geometric mean concentration at one month post-primary vaccination adjusted for country (pooled variance; Clin over Com group). PD = protein D. 95% CI = 95% confidence intervals (represented by the error bars). ^*^Immunological non-inferiority was demonstrated if the upper limit of the 95% confidence interval of the adjusted antibody GMC ratio (Clin over Com) was below 2.0.

In the primary vaccination phase, immune responses induced by the PHiD-CV Commercial lot were shown to be non-inferior to those induced by the Phase III Clinical lot (Figure 2; Additional file 1: Table S1).

One month post-primary vaccination, for each vaccine pneumococcal serotype, ≥96.3% and ≥93.6% of infants had antibody concentrations ≥0.2 μg/mL in the Clin and Com groups, respectively (Table 2). Antibody GMCs for serotypes 4 and 5 seemed higher in the Clin group. For each vaccine pneumococcal serotype, percentages of infants with OPA titres ≥8 were ≥95.6% in the Clin group, except for serotype 1, and ≥96.6% in the Com group, except for serotypes 1 and 6B (Table 3). OPA GMTs for serotype 18C seemed higher in the Clin group.

**Table 2:** **Immune responses to pneumococcal polysaccharides at one month post-primary vaccination (primary ATP immunogenicity cohort): 22F-ELISA**

	Clin group			Com group		
	N	% ≥0.2 μg/mL	GMC	N	% ≥0.2 μg/mL	GMC
		(95% CI)	(95% CI)		(95% CI)	(95% CI)
**Vaccine serotypes**						
Serotype 1	219	100	2.67	218	100	2.46
		(98.3 – 100)	(2.46 – 2.91)		(98.3 – 100)	(2.25 – 2.69)
Serotype 4	219	100	3.95	218	100	3.14
		(98.3 – 100)	(3.59 – 4.36)		(98.3 – 100)	(2.84 – 3.47)
Serotype 5	219	99.5	4.34	218	100	3.59
		(97.5 – 100)	(3.95 – 4.76)		(98.3 – 100)	(3.29 – 3.92)
Serotype 6B	219	96.3	1.31	218	93.6	1.23
		(92.9 – 98.4)	(1.16 – 1.48)		(89.5 – 96.4)	(1.07 – 1.41)
Serotype 7F	218	100	3.10	217	100	3.20
		(98.3 – 100)	(2.83 – 3.39)		(98.3 – 100)	(2.92 – 3.51)
Serotype 9V	219	100	3.34	218	100	3.14
		(98.3 – 100)	(3.03 – 3.69)		(98.3 – 100)	(2.83 – 3.49)
Serotype 14	219	99.5	5.13	218	100	4.74
		(97.5 – 100)	(4.54 – 5.79)		(98.3 – 100)	(4.23 – 5.32)
Serotype 18C	219	100	5.00	218	99.5	5.15
		(98.3 – 100)	(4.40 – 5.69)		(97.5 – 100)	(4.43 – 5.97)
Serotype 19F	219	99.5	6.69	218	99.5	6.96
		(97.5 – 100)	(6.04 – 7.41)		(97.5 – 100)	(6.26 – 7.73)
Serotype 23F	219	98.2	1.98	218	97.2	1.68
		(95.4 – 99.5)	(1.76 – 2.23)		(94.1 – 99.0)	(1.49 – 1.90)
**Cross-reactive serotypes**						
Serotype 6A	219	69.4	0.38	218	60.6	0.28
		(62.8 – 75.4)	(0.32 – 0.45)		(53.7 – 67.1)	(0.23 – 0.33)
Serotype 19A	219	61.6	0.27	218	54.6	0.23
		(54.9 – 68.1)	(0.23 – 0.31)		(47.7 – 61.3)	(0.20 – 0.27)

ATP = according to protocol.

Clin = group of infants from Malaysia and Singapore who received the Phase III Clinical lot of PHiD-CV in the primary vaccination phase.

Com = group of infants from Malaysia and Singapore who received the Commercial lot of PHiD-CV in the primary vaccination phase.

N = number of infants with available results.

95% CI = 95% confidence interval.

GMC = geometric mean concentration.

% = percentage of infants with antibody concentrations or OPA titres above the specified cut-off.

**Table 3:** **Immune responses to pneumococcal polysaccharides at one month post-primary vaccination (primary ATP immunogenicity cohort): OPA**

	Clin group			Com group		
	N	% ≥8	GMT	N	% ≥8	GMT
		(95% CI)	(95% CI)		(95% CI)	(95% CI)
**Vaccine serotypes**						
Serotype 1	209	88.5	128.9	210	90.0	122.1
		(83.4 – 92.5)	(102.7 – 161.7)		(85.1 – 93.7)	(98.3 – 151.7)
Serotype 4	207	100	698.3	207	98.1	609.3
		(98.2 – 100)	(619.6 – 786.9)		(95.1 – 99.5)	(519.6 – 714.3)
Serotype 5	207	98.6	127.9	210	96.7	98.6
		(95.8 – 99.7)	(109.0 – 149.9)		(93.3 – 98.6)	(83.0 – 117.1)
Serotype 6B	205	95.6	870.7	206	92.7	619.2
		(91.8 – 98.0)	(710.2 – 1067.6)		(88.3 – 95.9)	(483.4 – 793.2)
Serotype 7F	206	100	3905.8	208	100	3585.7
		(98.2 – 100)	(3420.2 – 4460.4)		(98.2 – 100)	(3119.8 – 4121.2)
Serotype 9V	207	100	1800.0	208	100	1851.3
		(98.2 – 100)	(1596.6 – 2029.3)		(98.2 – 100)	(1612.3 – 2125.8)
Serotype 14	209	99.5	1521.0	208	99.5	1485.8
		(97.4 – 100)	(1313.3 – 1761.6)		(97.4 – 100)	(1280.5 – 1724.0)
Serotype 18C	204	99.0	533.5	206	96.6	383.9
		(96.5 – 99.9)	(461.8 – 616.4)		(93.1 – 98.6)	(319.3 – 461.5)
Serotype 19F	206	98.1	689.6	206	97.1	573.5
		(95.1 – 99.5)	(581.1 – 818.2)		(93.8 – 98.9)	(477.2 – 689.3)
Serotype 23F	209	99.0	2716.7	207	99.5	2379.5
		(96.6 – 99.9)	(2316.3 – 3186.3)		(97.3 – 100)	(2043.4 – 2770.7)
**Cross-reacting serotypes**						
Serotype 6A	197	87.8	230.8	200	85.5	173.7
		(82.4 – 92.0)	(180.3 – 295.4)		(79.8 – 90.1)	(133.9 – 225.3)
Serotype 19A	197	42.1	18.1	199	37.7	15.1
		(35.1 – 49.4)	(13.7 – 23.8)		(30.9 – 44.8)	(11.5 – 19.8)

ATP = according to protocol.

OPA = opsonophagocytic activity.

Clin = group of infants from Malaysia and Singapore who received the Phase III Clinical lot of PHiD-CV in the primary vaccination phase.

Com = group of infants from Malaysia and Singapore who received the Commercial lot of PHiD-CV in the primary vaccination phase.

N = number of infants with available results.

95% CI = 95% confidence interval.

GMT = geometric mean titre.

% = percentage of infants with antibody concentrations or OPA titres above the specified cut-off.

For each vaccine pneumococcal serotype, ≥71.8% and ≥60.8% of toddlers at pre-booster vaccination, and ≥99.1% and ≥98.2% of toddlers one month post-booster vaccination, had antibody concentrations ≥0.2 μg/mL in the ClinCom and ComCom groups, respectively (Table 4).

**Table 4:** **Pre- and post-booster immune response to pneumococcal polysaccharides (ATP persistence and booster ATP immunogenicity cohorts)**

	ClinCom group						ComCom group					
	Pre-booster dose			Post-booster dose			Pre-booster dose			Post-booster dose		
	N	% ≥0.2 μg/mL	GMC	N	% ≥0.2 μg/mL	GMC	N	% ≥0.2 μg/mL	GMC	N	% ≥0.2 μg/mL	GMC
		(95% CI)	(95% CI)		(95% CI)	(95% CI)		(95% CI)	(95% CI)		(95% CI)	(95% CI)
**Vaccine serotypes**												
Serotype 1	111	85.6	0.48	107	100	7.14	112	75.0	0.35	111	100	6.29
		(77.6 – 91.5)	(0.40 – 0.57)		(96.6 – 100)	(6.12 – 8.32)		(65.9 – 82.7)	(0.30 – 0.41)		(96.7 – 100)	(5.38 – 7.35)
Serotype 4	107	86.9	0.56	106	100	7.53	106	82.1	0.48	109	100	7.43
		(79.0 – 92.7)	(0.48 – 0.67)		(96.6 – 100)	(6.44 – 8.80)		(73.4 – 88.8)	(0.40 – 0.58)		(96.7 – 100)	(6.33 – 8.71)
Serotype 5	103	92.2	0.76	106	100	7.91	103	91.3	0.54	108	100	7.16
		(85.3 – 96.6)	(0.65 – 0.89)		(96.6 – 100)	(6.91 – 9.06)		(84.1 – 95.9)	(0.47 – 0.63)		(96.6 – 100)	(6.25 – 8.20)
Serotype 6B	103	71.8	0.34	106	100	3.30	102	60.8	0.32	109	98.2	3.12
		(62.1 – 80.3)	(0.29 – 0.40)		(96.6 – 100)	(2.85 – 3.81)		(50.6 – 70.3)	(0.25 – 0.41)		(93.5 – 99.8)	(2.59 – 3.76)
Serotype 7F	104	95.2	0.88	106	100	9.02	105	96.2	0.91	109	100	9.25
		(89.1 – 98.4)	(0.75 – 1.03)		(96.6 – 100)	(7.77 – 10.47)		(90.5 – 99.0)	(0.78 – 1.07)		(96.7 – 100)	(8.04 – 10.64)
Serotype 9V	105	98.1	0.90	107	100	9.36	102	94.1	0.73	109	100	10.42
		(93.3 – 99.8)	(0.77 – 1.06)		(96.6 – 100)	(8.15 – 10.75)		(87.6 – 97.8)	(0.62 – 0.85)		(96.7 – 100)	(8.94 – 12.14)
Serotype 14	105	93.3	1.06	106	100	13.03	100	93.0	0.91	106	100	13.28
		(86.7 – 97.3)	(0.86 – 1.31)		(96.6 – 100)	(10.95 – 15.50)		(86.1 – 97.1)	(0.75 – 1.11)		(96.6 – 100)	(11.06 – 15.95)
Serotype 18C	109	92.7	0.83	106	100	19.80	108	92.6	0.78	108	100	24.19
		(86.0 – 96.8)	(0.69 – 1.01)		(96.6 – 100)	(17.02 – 23.03)		(85.9 – 96.7)	(0.65 – 0.93)		(96.6 – 100)	(20.66 – 28.33)
Serotype 19F	103	98.1	1.10	106	100	19.68	103	99.0	0.96	108	100	20.55
		(93.2 – 99.8)	(0.87 – 1.40)		(96.6 – 100)	(17.22 – 22.51)		(94.7 – 100)	(0.82 – 1.13)		(96.6 – 100)	(17.62 – 23.98)
Serotype 23F	108	83.3	0.66	107	99.1	7.19	106	79.2	0.47	109	100	6.83
		(74.9 – 89.8)	(0.51 – 0.84)		(94.9 – 100)	(5.94 – 8.71)		(70.3 – 86.5)	(0.38 – 0.58)		(96.7 – 100)	(5.77 – 8.07)
**Cross-reactive serotypes**												
Serotype 6A	109	53.2	0.23	106	99.1	2.13	111	45.0	0.21	108	97.2	1.99
		(43.4 – 62.8)	(0.18 – 0.28)		(94.9 – 100)	(1.70 – 2.66)		(35.6 – 54.8)	(0.16 – 0.26)		(92.1 – 99.4)	(1.60 – 2.49)
Serotype 19A	112	49.1	0.18	106	93.4	2.13	109	46.8	0.19	109	96.3	2.96
		(39.5 – 58.7)	(0.14 – 0.22)		(86.9 – 97.3)	(1.65 – 2.76)		(37.2 – 56.6)	(0.15 – 0.24)		(90.9 – 99.0)	(2.26 – 3.87)

ClinCom = group of toddlers from Singapore primed with the Phase III Clinical lot of PHiD-CV who received the Commercial lot of PHiD-CV in the booster vaccination phase.

ComCom = group of toddlers from Singapore primed with the Commercial lot of PHiD-CV who received the Commercial lot of PHiD-CV in the booster vaccination phase.

N = number of participants with available results.

95% CI = 95% confidence interval.

% = percentage of infants with antibody concentrations above the specified cut-off.

GMC = geometric mean concentration.

One month post-primary vaccination, for cross-reactive serotypes 6A and 19A, ≥60.6% and ≥54.6% of infants had antibody concentrations ≥0.2 μg/mL (Table 2), and ≥85.5% and ≥37.7% of infants had OPA titres ≥8, in the ClinCom and ComCom groups, respectively (Table 3). One month post-booster vaccination, for each cross-reactive serotype, ≥93.4% of toddlers reached antibody concentrations ≥0.2 μg/mL (Table 4).One month post-primary vaccination, ≥99.5% of infants had measurable anti-protein D antibodies and higher anti-protein D adjusted antibody GMCs were observed in the Clin group (Figure 2). The anti-protein D antibody seropositivity rates were 100% and 98.3% of toddlers at pre-booster vaccination, and 100% and 99.1% of toddlers at one month post-booster vaccination in the ClinCom and ComCom groups, respectively (data not shown).

#### Co-administered vaccine antigens

One month post-primary and post-booster vaccination, all children were seroprotected against diphtheria, tetanus and poliovirus types 1, 2 and 3, and all infants were seropositive for antibodies against the three pertussis antigens and polyribosylribitol phosphate from Hib. All infants except one were seroprotected against hepatitis B surface antigen (HBs) at one month post-primary vaccination. Three children had anti-HBs antibody concentrations which were initially measured between 10 and 100 mIU/mL. Out of these 3 children, upon retesting using *Immulite*™, one was found to be seroprotected while the other 2 had anti-HBs antibody concentrations <10 mIU/mL. This was further followed up by investigators and one child was subsequently re-vaccinated. Efforts to reach the other child for re-vaccination were unsuccessful. Three months after the second dose of the HRV vaccine, ≥81.9% of infants were seropositive for anti-HRV antibodies (data not shown).

### Safety

**Figure 3:**
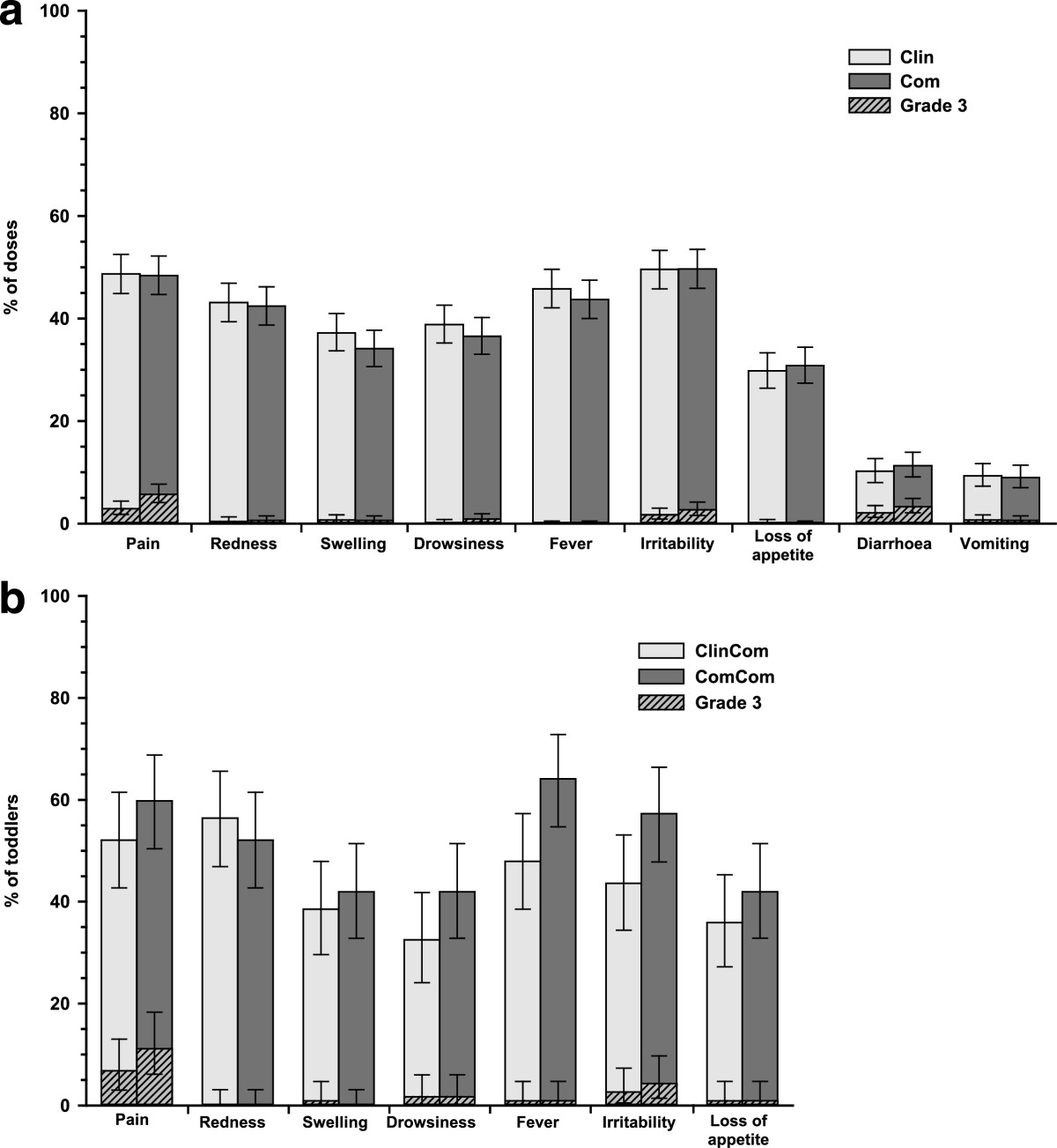
**Solicited symptoms following (a) primary vaccination (overall/dose; primary TVC) and (b) booster dose (booster TVC).** TVC = total vaccinated cohort. Clin = group of infants from Malaysia and Singapore who received the Phase III Clinical lot of PHiD-CV in the primary vaccination phase. Com = group of infants from Malaysia and Singapore who received the Commercial lot of PHiD-CV in the primary vaccination phase. ClinCom = group of toddlers from Singapore primed with the Phase III Clinical lot of PHiD-CV who received the Commercial lot of PHiD-CV in the booster vaccination phase. ComCom = group of toddlers from Singapore primed with the Commercial lot of PHiD-CV who received the Commercial lot of PHiD-CV in the booster vaccination phase. Error bars represent 95% confidence intervals. Solicited symptoms are recorded during 4-days post-vaccination.

In the primary vaccination phase, pain and diarrhoea were the most common overall per dose Grade 3 solicited local and general symptoms, respectively (Figure 3a). Diarrhoea was the most common Grade 3 solicited general symptom considered causally related to vaccination (following 15/698 and 22/698 doses in Clin and Com groups, respectively). Antipyretic use was reported after 296/698 and 276/699 doses, and antipyretics were given prophylactically after 45/698 and 41/699 doses, in the Clin and Com groups, respectively. During 31 days post-vaccination, 113/698 and 119/699 doses were followed by at least one unsolicited AE in the Clin and Com groups, respectively. The most frequently reported unsolicited AEs were upper respiratory tract infections (35/698 doses) and cough (10/698 doses) in the Clin group, and upper respiratory tract infections (42/699 doses) and pyrexia (7/699 doses) in the Com group. No Grade 3 unsolicited AEs were considered causally related to vaccination. SAEs were reported in 18 and 7 infants in the Clin and Com groups, respectively; none were considered causally related to vaccination and none were fatal.In the booster vaccination phase, the most common Grade 3 solicited local and general symptoms were pain and irritability, respectively (Figure 3b). Grade 3 solicited general symptoms considered causally related to vaccination were reported in ≤4.3% of toddlers, including Grade 3 fever in one toddler (0.9%) in each group. Antipyretic use was reported in 50/118 and 66/120 toddlers, and prophylactic antipyretics use in 7/118 and 4/120 toddlers, in the ClinCom and ComCom groups, respectively. Unsolicited AEs were reported in 18/118 and 25/120 toddlers in the ClinCom and ComCom groups, respectively. The most frequently reported unsolicited AEs were rhinorrhea (7/118 toddlers) and cough (5/118 toddlers) in the ClinCom group, and upper respiratory tract infections (6/120 toddlers) and rhinorrhea and pyrexia (each in 5/120 toddlers) in the ComCom group. One Grade 3 unsolicited AE considered causally related to vaccination (urticaria) was reported in the ComCom group. Four toddlers in the ComCom group reported SAEs; none were considered causally related to vaccination and none were fatal.

## Discussion

Three primary doses of PHiD-CV given to 2-, 3-, and 5-month-old infants from Malaysia and Singapore were shown to be immunogenic with a clinically acceptable safety profile. Non-inferiority of the immunogenicity of the PHiD-CV Commercial versus the Phase III Clinical lot was demonstrated for each vaccine pneumococcal serotype and protein D, although antibody GMCs for serotypes 4 and 5 and protein D, and OPA GMTs for serotype 18C, seemed higher in infants who received the Phase III Clinical lot. Nonetheless, as percentages of infants reaching threshold antibody concentrations and OPA titres for vaccine antigens were high and comparable in both groups, the observed differences in immunogenicity may be of limited clinical relevance. For each vaccine pneumococcal serotype, post-primary vaccination antibody GMCs seemed in line with those previously measured in Korea and Taiwan [16, 19], and higher than in Europe [15, 18, 21]. Although it remains unclear why the magnitude of immune responses to pneumococcal conjugate vaccines varies in different populations, plausible explanations include genetic factors, early exposure to *S. pneumoniae*, or nasopharyngeal carriage of pneumococcal serotypes [36]. Pre-vaccination antibody concentration, which is influenced by waning maternal antibodies and increasing adaptive immunity due to early exposure to *S. pneumoniae,* in Asian children was evaluated in a previous study conducted in Taiwan, where for each vaccine pneumococcal serotype the percentage of children with pre-vaccination antibody concentrations ≥0.2 μg/ml ranged from 11.5% to 42.5%, except serotype 14 (61%) [19]. Vaccine efficacy and effectiveness of pneumococcal conjugate vaccines have been demonstrated in various countries, hence clinical relevance of population differences remains unknown [37–42].

For each vaccine pneumococcal serotype, antibody GMCs calculated before the booster dose administration were lower than those measured one month post-primary vaccination, but ≥60.8% of toddlers from Singapore had pre-booster antibody concentrations ≥0.2 μg/mL. The booster dose of the PHiD-CV Commercial lot induced a robust immune response; virtually all toddlers reached antibody concentrations ≥0.2 μg/mL one month post-booster vaccination and antibody concentrations were higher than those measured post-primary vaccination, which suggested that primary vaccination of infants with PHiD-CV induced immunological memory [15, 16, 18, 21].

Primary vaccination with either vaccine lot and booster vaccination with the PHiD-CV Commercial lot induced immune responses against cross-reactive serotypes 6A and 19A. Some functional OPA responses for cross-reactive serotype 19A were observed following primary PHiD-CV vaccination, in-line with previous studies [15, 16, 18, 21]. However, the level of protection against serotype 19A conferred by the immune response induced by PHiD-CV should be further determined [43, 44].

Both PHiD-CV lots induced antibodies against the NTHi protein D carrier, which could potentially provide protection against disease caused by NTHi. Although a clear correlation between efficacy and anti-protein D antibody concentrations has not been established, efficacy trials with the predecessor 11-valent NTHi protein D-conjugated vaccine and PHiD-CV have suggested that the protein D carrier contributed to the induction of protection against acute otitis media due to NTHi [45, 46]. The co-administered vaccines given simultaneously with primary or booster doses of PHiD-CV were also immunogenic. This is consistent with other studies and suggests that no clinically relevant interference occurred between PHiD-CV and HRV or DTPa-based vaccines [17, 19].

No SAEs considered causally related to vaccination and no fatal SAEs were reported throughout the study. The reactogenicity and safety profiles of the Commercial and the Phase III Clinical lots of PHiD-CV were comparable and clinically acceptable, in line with previous studies [20].

The primary vaccination phase was powered to demonstrate the primary objectives. Other comparisons should be considered cautiously, since there was no adjustment for multiple comparisons of the various endpoints. The clinical relevance of the observed differences remains unknown, especially for antigens with no correlate of protection. The booster vaccination phase was also limited by its open design.

## Conclusions

This study showed that different lots of PHiD-CV were immunogenic with a clinically acceptable safety profile when given as 3-dose primary vaccination in 2-, 3- and 5-month-old infants from Malaysia and Singapore. A booster dose of the PHiD-CV Commercial lot induced a robust immune response in 18–21 month-old toddlers from Singapore. Both PHiD-CV lots were administered with other paediatric vaccines without compromising their immune response.

Synflorix, Infanrix-IPV/Hib, and Infanrix hexa are trademarks of the GlaxoSmithKline group of companies.

## Electronic supplementary material

Additional file 1: Between groups adjusted antibody GMC ratios post-primary vaccination (primary ATP immunogenicity cohort).(DOCX 17 KB)

Below are the links to the authors’ original submitted files for images.

Authors’ original file for figure 1

Authors’ original file for figure 2

Authors’ original file for figure 3

## Abbreviations

AE: Adverse event
ANOVA: Analysis of variance
ATP: According to protocol
CI: Confidence interval
DTPa-HBV-IPV/Hib: Diphtheria, tetanus, acellular pertussis-hepatitis B virus-inactivated poliovirus, and *H. influenzae* type b vaccine
ELISA: Enzyme-linked immunosorbent assay
GMC: Geometric mean concentration
GMT: Geometric mean titre
HRV: Human rotavirus
IPD: Invasive pneumococcal disease
OPA: Opsonophagocytic activity
PHiD-CV: Pneumococcal non-typeable *Haemophilus influenzae* (NTHi) protein D conjugate vaccine
SAE: Serious adverse event.
