# Supplementary material for: A randomised trial to evaluate the immunogenicity, reactogenicity, and safety of the 10-valent pneumococcal non-typeable Haemophilus influenzaeprotein D conjugate vaccine (PHiD-CV) co-administered with routine childhood vaccines in Singapore and Malaysia
Source: BMC Infect Dis. 2014 Oct 2;14:530. doi: 10.1186/1471-2334-14-530 (PMC4286912; doi:10.1186/1471-2334-14-530)
Supplement: Supplementary file 1 — Additional file 1: Between groups adjusted antibody GMC ratios post-primary vaccination (primary ATP immunogenicity cohort).(DOCX 17 KB) [file 12879_2014_3854_MOESM1_ESM.docx]

**Supplementary Table 1** Between groups adjusted antibody GMC ratios post-primary vaccination (primary ATP immunogenicity cohort).

|  | | | | | **Ratio of adjusted GMCs   (Clin / Com )** | | |
| --- | --- | --- | --- | --- | --- | --- | --- |
|  | **Clin** | | **Com** | |  | **95% CI** | |
| **Antibody** | **N** | **Adjusted GMC** | **N** | **Adjusted GMC** | **Value** | **LL** | **UL** |
| ANTI-1 | 219 | 2.68 | 218 | 2.47 | 1.09 | 0.96 | **1.23** |
| ANTI-4 | 219 | 3.94 | 218 | 3.14 | 1.26 | 1.09 | **1.45** |
| ANTI-5 | 219 | 4.31 | 218 | 3.57 | 1.21 | 1.06 | **1.37** |
| ANTI-6B | 219 | 1.30 | 218 | 1.22 | 1.07 | 0.88 | **1.28** |
| ANTI-7F | 218 | 3.06 | 217 | 3.16 | 0.97 | 0.85 | **1.10** |
| ANTI-9V | 219 | 3.32 | 218 | 3.12 | 1.06 | 0.92 | **1.23** |
| ANTI-14 | 219 | 4.99 | 218 | 4.63 | 1.08 | 0.91 | **1.27** |
| ANTI-18C | 219 | 5.03 | 218 | 5.17 | 0.97 | 0.80 | **1.18** |
| ANTI-19F | 219 | 6.69 | 218 | 6.95 | 0.96 | 0.83 | **1.11** |
| ANTI-23F | 219 | 1.98 | 218 | 1.68 | 1.18 | 0.99 | **1.40** |
| ANTI-PD | 219 | 2591.82 | 218 | 1901.14 | 1.36 | 1.18 | **1.58** |

Footnote: Clin= group of infants from Malaysia and Singapore who received the Phase III Clinical lot of PHiD-CV in the primary vaccination phase

Com= group of infants from Malaysia and Singapore who received the Commercial lot of PHiD-CV in the primary vaccination phase

N= number of participants with available results

95% CI= 95% confidence interval

LL= Lower limit; UL= Upper limit

Adjusted GMC ratio= geometric mean concentration adjusted for country (pooled variance; Clin over Com group)
