# Supplementary figures and images for: A randomised trial to evaluate the immunogenicity, reactogenicity, and safety of the 10-valent pneumococcal non-typeable Haemophilus influenzaeprotein D conjugate vaccine (PHiD-CV) co-administered with routine childhood vaccines in Singapore and Malaysia
Source: BMC Infect Dis. 2014 Oct 2;14:530. doi: 10.1186/1471-2334-14-530 (PMC4286912; doi:10.1186/1471-2334-14-530)

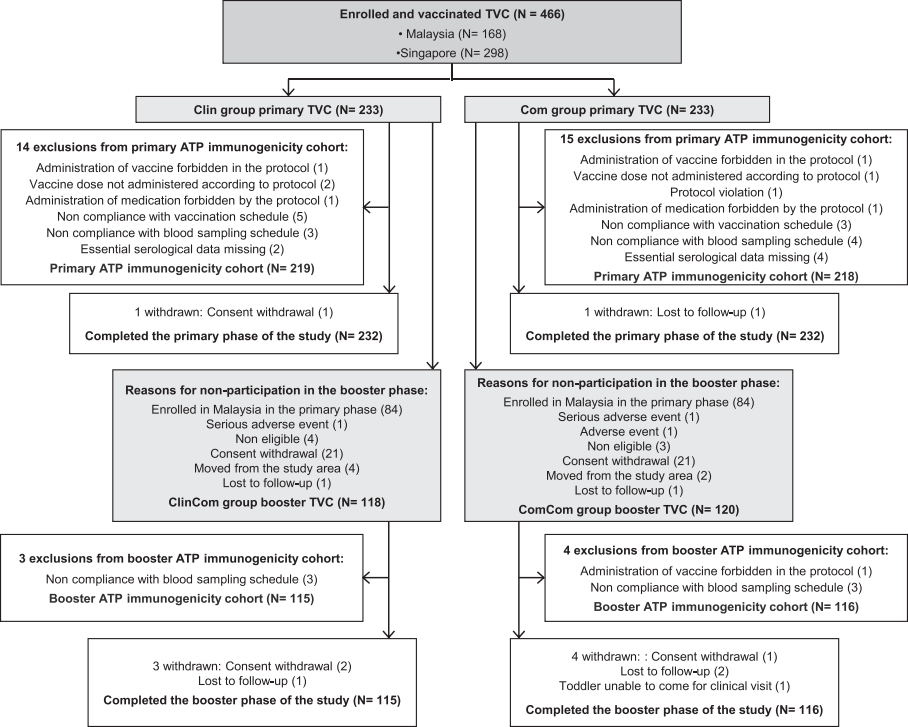

Supplement: Supplementary file 2 — Authors’ original file for figure 1 [file 12879_2014_3854_MOESM2_ESM.pdf]

Antigen

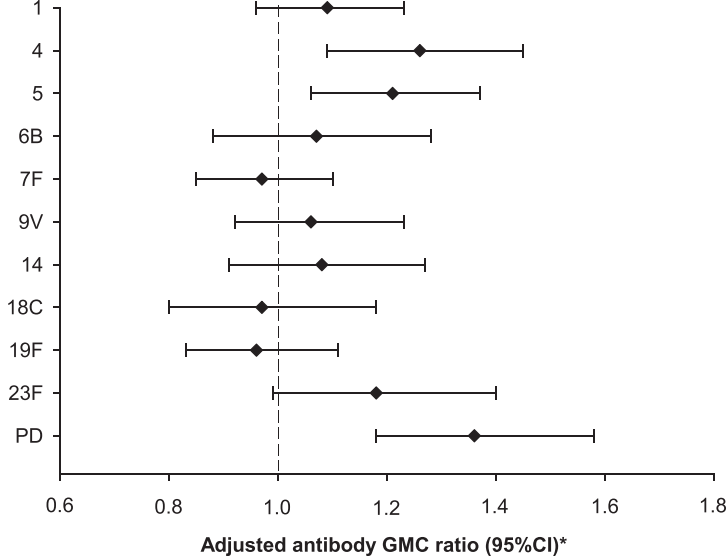

Supplement: Supplementary file 3 — Authors’ original file for figure 2 [file 12879_2014_3854_MOESM3_ESM.pdf]

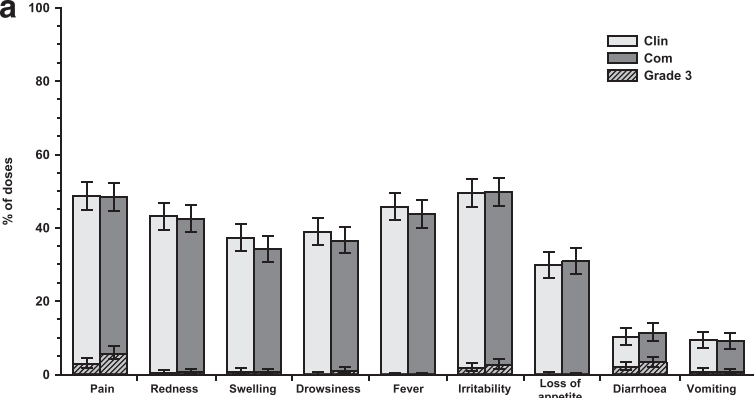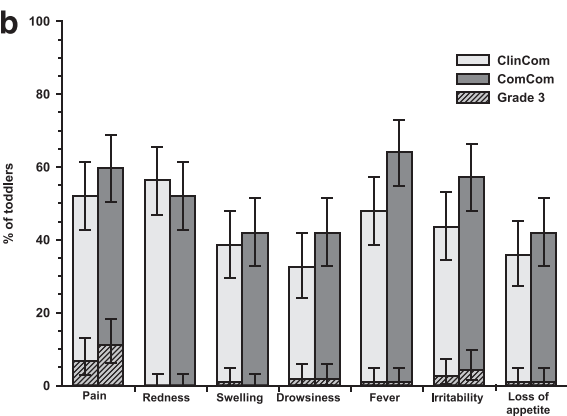

Supplement: Supplementary file 4 — Authors’ original file for figure 3 [file 12879_2014_3854_MOESM4_ESM.pdf]
